# Facilitated transitions: coaching to improve the medical school to residency continuum

**DOI:** 10.1080/10872981.2020.1856464

**Published:** 2020-12-02

**Authors:** Margaret Wolff, Paula Ross, Jasmyne Jackson, Eric Skye, Tamara Gay, Margaret Dobson, David T. Hughes, Helen Kang Morgan

**Affiliations:** a Emergency Medicine and Pediatrics, University of Michigan Medical School, Ann Arbor, MI, USA; b RISE, University of Michigan Medical School, Ann Arbor, MI, USA; c Pediatrics, Boston Combined Residency Program, Boston, MA, USA; d Family Medicine, University of Michigan Medical School, Ann Arbor, MI, USA; e Psychiatry, University of Michigan Medical School, Ann Arbor, MI, USA; f Surgery, University of Michigan Medical School, Ann Arbor, MI, USA; g Department of Obstetrics and Gynecology and Learning Health Sciences, University of Michigan, Ann Arbor, MI, USA

**Keywords:** Mentorship, coaching, undergraduate medical education, graduate medical education

## Abstract

**Background**: Current efforts incompletely address the educational, social, and developmental aspects of a learner’s transition from medical school to residency.

**Objective**: To determine the feasibility and acceptability of a transition to residency (TTR) coaching program.

**Designs**: In March 2019, we designed, implemented, and evaluated a TTR coaching program for students who matched into residency programs at our institution. Goals were to stimulate reflection on successes and challenges encountered during medical school, develop strategies to problem-solve barriers and address concerns, identify professional and personal resources, improve confidence, and make an action plan.

**Results**: Of eligible learners, 42% (10/24) enrolled in TTR coaching. Learners were most interested in coaching in the following areas: wellbeing (70%, 7/10), interpersonal/communication skills (60%, 6/10), and learning plan development (50%, 5/10). The majority (90%; 9/10) expressed satisfaction with the program and would recommend participation. One month after starting internship, 90% (9/10) of learners stated the program helped facilitate their transition. Learners who did not enroll in TTR cited concerns around the coach selection process (72%, 8/11), upcoming travel (45%, 5/11), insufficient time/competing demands (27%, 3/11), and lack of perceived benefit (18%, 2/11).

**Conclusion**: This pilot study demonstrated preliminary feasibility and acceptability for TTR coaching.

## Introduction

The transition from undergraduate medical education (UME) to graduate medical education (GME) is an exciting, but potentially angst-ridden time for trainees[1]. Efforts are underway to improve both trainee preparation and UME to GME communication at this transition[2]. While these initiatives address the continuity of learning, these efforts fail to fully consider the educational, social, and developmental aspects of transitions in medical education[3]. These initiatives perpetuate the learner’s reliance on external forces to drive their learning instead of using this transition point to foster professional identity formation and the development of self-directed learners[4]. From a developmental perspective, this transition is a critically intensive learning period and initiatives should be individualized to each learner’s developmental trajectory [5,6].

Coaching has been described as an effective method to individualize training and support learner development through gap identification, goal creation, and personal accountability[7]. There is strong evidence to support coaching in medical education for technical skills and growing evidence to support coaching for wellbeing, resilience, and improved non-technical skills[8]. A coach facilitates learner development by prompting reflection, clarifying relevant outcomes, and assisting with the identification of specific actions needed to achieve the desired goals [7,8]. Despite the clear alignment of the goals of coaching and transition to residency initiatives, coaching has not been described in the transition to residency phase. Drawing on the coaching literature, we designed, implemented, and evaluated a learner-driven, transition to residency (TTR) coaching program for recently matched fourth-year medical students. The primary goals of this pilot program were to ease the transition to residency while supporting their development into self-directed learners. We sought to determine the feasibility and acceptability of the TTR coaching program.

## Methods

Seven faculty (six physicians from emergency medicine, obstetrics, and gynecology, primary care, psychiatry, and surgery) and one non-physician (PhD in medical sociology) were trained as TTR coaches through a three-day structured, professional coach certificate program. Training covered the foundation of coaching practices, coaching skills (e.g., creating a contract, utilizing questioning) and introduction to various resources (e.g., mind-mapping, 360 degree evaluations)[7]. Activities included didactics, small group discussion, role play and culminated in simulated coaching with immediate feedback.

Following the March 2019 National Resident Matching Program, all students at our institution who matched into a residency program at our institution were invited to participate. These students had all previously participated in coaching through a mandatory UME coaching program at our institution that begins in the first year and ends with residency selection. Students were introduced to transition coaching in an introductory session. In addition, students were introduced to the Master Adaptive Learner (MAL) framework as the model for self-directed, adaptive learning to strive for as they began the coaching program[9]. The MAL framework is an iterative four phased-process based on self-directed learning theory: Planning, Learning, Assessing, and Adjusting[9]. After consenting to participate, students were provided a brief written introduction to each coach outlining their coaching style. Students did not have prior formal relationships with the coaches. Students then ranked their coach preference and were matched with a coach. Goals were to stimulate reflection on successes and challenges encountered during medical school, develop strategies to problem-solve barriers and address concerns, identify professional and personal resources, improve confidence, and make an action plan. Coaches were granted access to their coachees’ UME performance data to utilize as the basis for reflection and discussion. Students met with their assigned coaches between March – July 2019; three sessions were recommended. Table 1 outlines session goals, however, the format and content were flexible based on individual student needs.

**Table 1.** Transition to residency coaching program structure

Session	Objective(s)
Session 1	Contract and review of basic ground rules – number of sessions, timing, confidentiality Timeline sharing (coach goes first, then student shares) Discuss student’s goals of participation
Homework	Coach – review student medical school performance data with focus on the goals the student selected Data sources – medical school performance data (including clinical evaluation forms, residency preparation course data) Student – start to think about potential goals
Session 2	Review feedback Introduce goal setting SMART goals Set 2–3 goals
Homework	Refine goals if needed (and email coach) Start taking action on goals
Session 3	Review what action(s) have been taken on goal Explore barriers Explore what enabled success (for taking action on goal) Brainstorm how to sustain an action plan (in general or specifically for these goals) as internship starts How to find coaching in internship Brief exit survey

Students completed a pre-participation survey assessing rationale for participating. Students that did not enroll were surveyed to explore reasons. Both students and coaches completed a survey regarding the feasibility and acceptability of the program; students were also asked about the domains covered. One month after starting internship, learners were surveyed regarding progress on goals. All surveys were conducted via an online platform. Questions were developed by study team and piloted by GME trainees with experience with coaching. Surveys were multiple choice with options for free text. Free text was coded and categorized by the study team. This project was determined to be exempt by our Institutional Review Board.

## Results

### Participants

Of the 24 eligible students, 42% (10/24) enrolled. Participants represented diverse specialties. Prior to starting, students cited the following reasons for enrolling: help brainstorming wellness strategies (90%, 9/10), address worries about internship (70%, 7/10), create goals (70%, 7/10), develop an action plan (60%, 6/10), problem solve barriers to achieving goals (60%, 6/10), identify resources (50%, 5/10) and build confidence (40%, 4/10). Students reported creating goals in these areas: wellbeing (70%, 7/10), interpersonal/communication skills (60%, 6/10), learning plan development (50%, 5/10), medical knowledge (30%, 3/10), and professionalism (20%, 2/10). One month into intern year, (50%, 5/10) of learners had taken action on their goals.

### Acceptability

The majority of participants (90% of learners, 9/10; 100% of coaches) perceived the program to be highly valuable, with 90% (9/10) of learners stating they recommend the program. Although all participants expressed satisfaction with their coaches (10/10, 100%), half (50%; 5/10) would have preferred to continue with their UME coach and 20% (2/10) preferred a coach in their specialty. When contacted 1 month into intern year, 90% (9/10) of learners stated the program helped facilitate their transition.

### Feasibility

All students met with their coach at least once, 90% (9/10) had two meetings, 40% (4/10) had three meetings, and 20% (2/10) had four meetings. Meetings were in person (72%, 18/25), via an online platform (24%, 6/25), or via telephone (4%, 1/25). Most coaches reported scheduling difficulty (87%, 7/8). Email was used to summarize meetings, provide accountability, or refine goals, and provide feedback (71%, 5/7). Coaches reported the lack of actionable performance data from medical school (86%, 6/7) as the biggest challenge to facilitating meaningful discussion. This was in part due to the limited clinical rotations students participated in during the fourth year.

### Feedback from non-participants

A large majority of students (79%; 11/14) who did not enroll provided feedback. They cited concerns around coach selection (72%, 8/11), travel (45%, 5/11), insufficient time/competing demands (27%, 3/11), and lack of perceived benefit (18%, 2/11). Those concerned about coach selection stated they would have considered participating if they could have continued working with their UME coach (62%, 5/8) or were guaranteed a coach in their specialty (38%, 3/8).

## Discussion

This brief coaching intervention was feasible with high acceptability among participants. As coaching programs are increasingly being implemented at medical schools around the country, our pilot intervention illustrates how coaching can benefit learners at the vital medical school to residency transition.

The overarching goal of the program was to ease the transition to residency for the participants. The ways in which this was actualized for participants varied. A key part of this program was the individualized focus allowing learners to identify and pursue what was important to them during this transition. This approach is consistent with the national call to individualize this transition and is congruent with the premise of coaching [5,7]. The diverse goals learners created provide insights into the areas students find most valuable during this time-limited space. It is striking that many chose to work with their coach on wellbeing. Research has demonstrated that burnout and emotional exhaustion increase in residency with the highest rates of resident suicides occurring within the first 3 months of internship [10,11]. There is limited evidence to support any one particular approach to increasing wellbeing during this time period. However, there is preliminary evidence to support using coaching to promote wellbeing for practicing physicians[8]. Further research is needed to determine the efficacy of coaching on learner wellbeing during this transition.

While this program was beneficial for participants, the biggest challenge was our inability to enlist all eligible learners. This consistent with the trend of the fourth year of medical school often being under-utilized by learners [12,13]. Starting post-match day likely contributed; learners indicated they planned to use this time to travel, spend time with loved ones, find new housing, or experience major life events such as getting married. To address this challenge, and given the success of this project, we are expanding TTR coaching to all students (not only those who match at our institution) by incorporating TTR coaching into the existing longitudinal UME coaching program. TTR coaching will begin earlier in the fourth year with students’ coach assigned during their first year. Currently, our academic coaching program focuses on development during the first and second years with minimal interaction in the third and fourth years. The expansion of the program will allow all students to participate in TTR coaching and encourage students to begin addressing many of their concerns and gaps earlier. This will require targeted training on transition-specific topics for all of our academic coaches.

A notable challenge our coaches faced was the paucity of quality performance data to help learners identify recurring themes to create actionable goals. As UME programs move towards competency-based education, educators must keep the need for quality performance data at the forefront of their evaluation and assessment programs.

## Limitations

This intervention is primarily limited by the small sample size and single-center approach. In addition, students ranked their coach preference which may not be feasible when expanded to a larger scale.

## Conclusion

TTR coaching fills a gap in the UME to GME transition for individualized support. TTR coaching is both feasible and acceptable and creates space for students to focus on their clinical development and wellness goals.
